# The effect of bariatric surgery in comparison with the control group on the prevention of comorbidities in people with severe obesity: a prospective cohort study

**DOI:** 10.1186/s12893-022-01740-7

**Published:** 2022-07-28

**Authors:** Amir Ebadinejad, Maryam Barzin, Behnaz Abiri, Maryam Mahdavi, Alireza Khalaj, Danial Ebrahimi, Farhad Hosseinpanah, Majid Valizadeh

**Affiliations:** 1 Obesity Research Center, Research Institute for Endocrine Sciences, Shahid Beheshti University of Medical Sciences, Tehran, Iran; 2 Tehran Obesity Treatment Center, Department of Surgery, Faculty of Medicine, Shahed University, Tehran, Iran; 3 Department of Surgery, Faculty of Medicine, Shiraz University, Shiraz, Iran

**Keywords:** Bariatric surgery, Diabetes, Hypertension, Dyslipidemia

## Abstract

**Background:**

Obesity is a global health priority, particularly in developing countries. The preventive effect of bariatric surgery against obesity-related diseases in the developing countries of the Middle East and North Africa region, where type 2 diabetes mellitus (T2DM), hypertension (HTN), and dyslipidemia prevail, has not been examined.

**Method:**

Severely obese participants who underwent bariatric surgery were compared with their counterparts who underwent no intervention. These patients had been followed up in two prospective cohort studies for three years. We here determined the incidence of new-onset T2DM, HTN, and dyslipidemia and reported absolute and relative risks for the incidence of these comorbidities in the two groups.

**Results:**

In this study, 612 participants in the bariatric surgery group were compared with 593 participants in the control group. During the follow-up period, T2DM developed in eight (2.9%) people in the surgery group and 66 (15.0%) people in the control group (*P* < 0.001). New-onset HTN and dyslipidemia showed significantly lower frequencies in the surgery group compared to the control group (4 (1.8%) vs. 70 (20.4%) and 33 (14.3%) vs. 93 (31.5%), respectively). Regarding a less favorable metabolic profile in the surgery group at the baseline, the relative risk reductions associated with bariatric surgery were 94, 93, and 55% for the development of T2DM, HTN, and dyslipidemia, respectively.

**Conclusion:**

The risk reduction of obesity-related comorbidities after bariatric surgery should be considered in the decision-making process for public health in the region, which bariatric surgery could result in the prevention of comorbidities.

**Supplementary Information:**

The online version contains supplementary material available at 10.1186/s12893-022-01740-7.

## Background

Obesity is the second leading cause of preventable premature death [1]. Mean worldwide body mass index (BMI) is steadily on the rise, and current trends predict that 20% of the global population will be classified as obese by 2030 [2]. In Iran, obesity prevalence is 15.6% in men and 30.4% in women, and the rate of severe obesity among Tehranian men and women, with an increasing trend, reached 4.5% and 10.9% in 2016, respectively. [3, 4]. Obesity is an established risk factor for overall all-cause mortality and obesity-related comorbidities, including type 2 diabetes mellitus (T2DM), hypertension (HTN), and dyslipidemia [5, 6].

Therapeutic options for severe obesity include non-surgical treatments and bariatric surgery. The non-surgical approach usually encompasses a multicomponent strategy comprising behavioral therapy, dietary recommendations, increased physical activity, and pharmacotherapies, which often fails [7, 8]. Notable recent studies, however, indicate that pharmacological therapy for optimal weight loss in overweight or obese patients offers promising results [9–11]. Compared with the non-surgical treatment of obesity, bariatric surgery offers a safe, efficient, and permanent approach for weight loss and delivers higher remission rates for T2DM and metabolic syndrome [8, 12, 13]. Compared to non-surgical and pharmaceutical treatments, bariatric surgery has shown protective effects against obesity-related diseases, which is related to significant weight loss and physiological changes [14]. A recent meta-analysis reported that bariatric surgery was associated with the relative risk reductions of 61%, 64%, and 77% for the development of T2DM, HTN, and dyslipidemia, respectively; however, the number of studies was insufficient in the recent meta-analysis, and all the studies included had been conducted in developed countries [15]. To our knowledge, no study has examined the preventive effects of bariatric surgery against obesity-related diseases in the developing countries located in the Middle East and North Africa region (MENA), where T2DM, HTN, and dyslipidemia prevail and people have less tendency for bariatric surgery compared to other communities [16, 17]. In this cohort study, we examined the preventive effects of bariatric surgery against obesity-associated T2DM, HTN, and dyslipidemia over a mid-term follow-up.

## Methods and materials

### Study design and participants

**Fig. 1:**
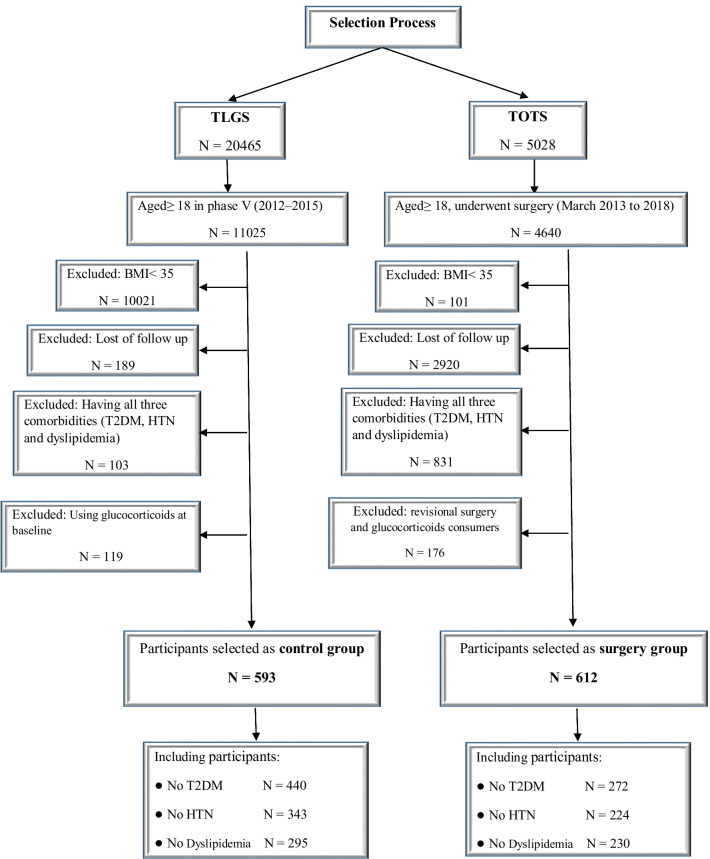
The participant selection process for the surgery and control groups

In this study, we prospectively investigated the incidence of new-onset comorbidities in two cohorts of people living in Tehran during a specific period of time. As the control group, individuals with severe obesity and over 18 years of age were included from a population-based cohort, the Tehran Lipid, and Glucose Study (TLGS). As the surgery group, individuals with severe obesity and over 18 years of age participating in another ongoing cohort, the Tehran Obesity Treatment Study (TOTS), who underwent bariatric surgery for the first time were included. All the individuals selected from the two studies were in the same time period and lived in the same state. Those who were under treatment with glucocorticoids were excluded. Patients who initially had all three T2DM, HTN, and dyslipidemia were excluded. In order to evaluate the incidence of one comorbidity, patients who did not initially have it in each group were followed up. Therefore, the sum of participants without one comorbidity is greater than the total number because some patients may have two comorbidities from the beginning. The participant selection process in the two groups has been illustrated in Fig. 1.

### TLGS

TLGS is a prospective study initiated in 1999 as a large-scale community-based study aiming to determine the prevalence of non-communicable disease risk factors in a representative sample of the residents of District 13 of Tehran, the capital city of Iran. Its data are collected prospectively at 3-year intervals. The sampling and follow-up methods have been described previously [18]. Among 11,025 eligible individuals entering phase V of the study (2011–2014) and continuing to phase VI (2015–2018), after applying the exclusion criteria, 593 people with a mean age of 49.0 years and a BMI of 38.6 kg/m^2^ were finally selected.

### TOTS

TOTS is an ongoing single-institution prospective study initiated in March 2013, in which the participants undergoing a bariatric procedure based on an individualized clinical decision plan are registered. The participants entering TOTS are referred to one of three university-affiliated hospitals for clinical assessments and surgical procedures. All TOTS participants undergo bariatric surgery, including sleeve gastrectomy (SG), one-anastomosis gastric bypass (OAGB), and Roux-en-Y gastric bypass (RYGB). More information regarding the study’s protocol is available elsewhere [19]. Out of 4640 eligible individuals, we finally included 612 participants from March 2013 to March 2019, with a mean age of 39.2 years and a BMI of 45.0 kg/m^2^ (364 cases of SG, 210 cases of OAGB, and 38 cases of RYGB).

The incidence of new-onset T2DM in both surgical and control groups was evaluated in participants who did not have T2DM at baseline (272 vs. 440). Also, participants without HTN (224 vs. 343) and dyslipidemia (230 vs. 295) at the beginning of the study were evaluated for the incidence of new-onset HTN and dyslipidemia.

The Ethics Committee of Research Institute for Endocrine Sciences, Shahid Beheshti University of Medical Sciences, Tehran, Iran, approved all procedures concerning human participants (IR.SBMU.ENDOCRINE.REC. 1400.050). This study was performed in accordance with the ethical standards of the Declaration of Helsinki (1964) and its later amendments or comparable ethical guidelines. Written informed consent was obtained from all the participants included in the study.

### Clinical and biochemical measurements

The details of data collection (demographic, laboratory, and clinical) at the baseline and follow-ups in the two cohort studies (i.e., TLGS and TOTS), which followed a somewhat similar process, have been precisely described [18, 19].

### Definitions of terms

Type 2 diabetes mellitus was defined based on the American Diabetes Association’s criteria as having either fasting plasma glucose (FPG) levels ≥ 126 mg/dL, 2-h plasma glucose (2-hPG) ≥ 200 mg/dL, or HbA1c > 6.5% or being under treatment with hypoglycemic drugs [20]. HbA1c was not available for the participants of TLGS, hence FPG and 2-hPG levels were utilized; for the surgical group selected from TOTS, laboratory FPG and HbA1c data were available, which we used to diagnose T2DM. Regarding HTN, the subjects who had systolic BP levels higher than 140 mmHg or diastolic BP levels of 90 mmHg or consumed antihypertensive medications were considered hypertensive [21]. Dyslipidemia was defined as either TC > 240 mg/dL, HDL < 40 mg/dL, TG > 200 mg/dL, or LDL > 160 mg/dL or being under treatment with lipid-lowering drugs [22].

### Statistical procedures

All normally-distributed continuous variables were expressed as mean ± standard deviation (SD). Otherwise, skewed continuous variables were reported as the median and interquartile range (IQR) 25–75%. Baseline categorical variables were expressed using frequency (percentage). Differences in various characteristics between the control and surgery groups were tested using the independent sample t-test, Mann–Whitney U test, and chi-square test for normal, skewed, and categorical variables, respectively. To assess possible differences between the study groups, binary logistic regression was applied. Possible confounders (age, sex, BMI, and a family history of T2DM at the baseline) were included in the model. All statistical analyses were performed using STATA version 12 (STATA, College Station, TX, USA); the significance level was set at P < 0.05 (two-tailed).

## Results

Participants with severe obesity were enrolled from two cohort studies (i.e., TOTS that was the source for the surgery group’s participants and TLGS that provided the participants of the control group). Baseline characteristics of participants in the surgery and control groups have been demonstrated in Table 1. Female participants constituted 503 (82.2%) in the surgery group and 468 (78.9%) in the control group. Anthropometric parameters, including weight, BMI, and waist, and hip circumferences, were significantly higher in the surgery group. Baseline characteristics of participants in the surgery and control groups based on the absence of T2DM or HTN or dyslipidemia at baseline have been demonstrated in Additional file 1: Table S1.

**Table 1:** Baseline Characteristics of Study Participants

Characteristic	Surgery group N = 612	Control group N = 593	*P* value
Female n (%)	503 (82.2)	468 (78.9)	0.152
Age (year)	39.2 ± 10.9	49.0 ± 13.3	< 0.001
Weight (kg)	119.8 ± 20.1	96.5 ± 15.5	< 0.001
BMI (kg/m^2^)	45.0 ± 6.0	38.5 ± 3.5	< 0.001
BMI < 40, n (%)	106 (17.3)	434 (73.2)	< 0.001
BMI 40–50, n (%)	407 (66.5)	155 (26.1)	
BMI > 50, n (%)	99 (16.2)	4 (0.7)	
Waist circumference (cm)	123.4 ± 13.9	113.4 ± 10.2	< 0.001
Hip circumference (cm)	135.8 ± 12.6	116.3 ± 8.3	< 0.001
FPG (mg/dl)	106.5 ± 32.5	101.5 ± 18.3	0.001
HbA1c %	5.6 ± 1.0	NA	NA
2-hPG (mg/dl)	NA	123.1 ± 38.5	NA
Impaired fasting glucose n (%)	223 (48.6)	217 (43.4)	0.108
T2DM n (%)	123 (21.4)	84 (14.7)	< 0.001
Systolic BP (mm Hg)	123.9 ± 13.9	125.3 ± 18.3	0.123
Diastolic BP (mm Hg)	79.5 ± 8.8	82.1 ± 10.6	< 0.001
HTN, n (%)	169 (29.1)	245 (41.5)	< 0.001
Total cholesterol (mg/dl)	190.1 ± 36.4	198.8 ± 38.1	< 0.001
Triglycerides (mg/dl)	138.0 (102.0–181.2)	148.0 (112.0–192.5)	0.006
HDL cholesterol (mg/dl)	49.4 ± 12.4	48.3 ± 11.4	0.112
LDL cholesterol (mg/dl)	109.9 ± 32.3	118.0 ± 33.1	< 0.001
Dyslipidemia n (%)	274 (47.4)	295 (49.8)	0.406
Never smoking n (%)	483 (83.7)	525 (89.3)	0.003
Family history of T2DM n (%)	218 (47.7)	69 (11.8)	< 0.001

*BMI* body mass index, *FPG* fasting plasma glucose, *2-hPG* 2-h plasma glucose, *T2DM* type 2 diabetes mellitus, *BP* blood pressure, *HTN* hypertension

Data are presented as mean ± SD or n (%) except triglycerides which are presented as median (IQ 25–75)

**Fig. 2:**
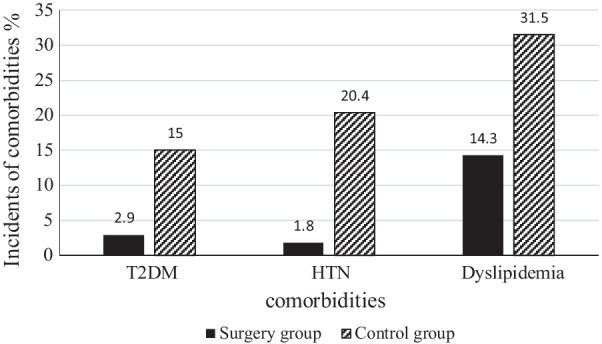
The incidence rates of new-onset comorbidities in the participants at the end of the follow‑up period (p-value < 0.001 for all comorbidities)

The median durations of the follow-up in the surgery and control groups were 3.63 (2.82–5.08) and 3.21 (2.93–3.69) years, respectively. Anthropometric measures showed significant variations between the groups at the follow-up (*P* < 0.001). In the surgery group, weight and BMI reached from 119.8 (20.1) kg and 45.0 (6.0) kg/m^2^ to 81.9 (14.2) kg and 30.8 (4.6) kg/m^2^, respectively, whereas in the control group, they reached from 96.55 (15.6) kg and 38.56 (3.5) kg/m^2^ to 95.9 (17.0) kg and 38.0 (4.7) kg/m^2^, respectively. New-onset comorbidities in the participants of the two groups during the follow-up have been shown in Fig. 2. During the follow-up, T2DM developed in 8/272 (2.9%) participants of the surgery group (3 patients with consuming medications and 5 patients with impaired HbA1C and FPG) and 66/440 (15.0%) participants of the control group (35 patients with consuming medications and 9 patients with impaired 2-hPG and FPG and 22 patients with impaired 2-hPG or FPG). (New-onset HTN and dyslipidemia were significantly lower in the surgery group vs. the control group (4/224 (1.8%) vs. 70/343 (20.4%) and 33/230 (14.3%) vs. 93/295 (31.5%), respectively). There was no significant difference between the three types of surgical methods regarding the incidence of new-onset comorbidities.

Absolute risk (AR) and relative risk (RR) for new-onset comorbidities have been reported in Table 2. The participants undergoing surgery compared to the control group had a lower overall risk for new-onset T2DM (AR = 2.9% vs. 15.0%; absolute risk reduction (ARR) = 12.0% [95% CI: 8.1–15.9%]; adjusted-RR = 0.06 [95% CI: 0.02–0.17]), HTN (AR = 1.7% vs. 20.4%; ARR = 18.6% [95% CI: 14.2–23.2%]; adjusted-RR = 0.07 [95% CI: 0.02–0.22]), and dyslipidemia (AR = 14.3% vs. 31.5%; ARR = 17.1% [95%CI: 10.2–24.1%]; RR = 0.45 [95%CI: 0.26–0.78]). According to ARR, the “number need to treat” (NNT) for T2DM, was estimated at 8 (95% CI, 6–12), indicating that by surgery of 8 cases, 1 case of T2DM would be treated; also NNT for HTN, and dyslipidemia was estimated at 5 (95% CI, 4–7) and 5 (95% CI, 4–9), respectively.

**Table 2:** Absolute and relative risks for new-onset comorbidities

	% (95% CI)						
	Absolute risks surgery group	Absolute risks control group	Absolute risk reduction	Unadjusted relative Risk	Adjusted^a^ relative Risk		Number need to treat
New-DM n (%)	2.9 (0.9–4.9)	15.0 (11.6–18.3)	12.0 (8.1–15.9)	0.17 (0.08–0.36)	0.06 (0.02–0.17)		8 (12–6)
New-HTN n (%)	1.7 (0.5–3.5)	20.4 (16.1–24.6)	18.6 (14.2–23.2)	0.07 (0.02–0.20)	0.07 (0.02–0.23)		5 (7–4)
New-Dyslipidemia n (%)	14.3 (9.8–18.8)	31.5 (26.2–36.8)	17.1 (10.2–24.1)	0.36 (0.23–0.57)	0.45 (0.26–0.78)		5 (9–4)

*T2DM* type 2 diabetes mellitus, *HTN* hypertension, *CI* confidence interval

^a^The analyses were adjusted for sex, age, and body mass index at baseline

## Discussion

In this prospective study on the severe obese individuals taking part in two population-based cohorts (.e., TLGS and TOTS), the participants who underwent bariatric surgery had significantly lower probability to develop T2DM, HTN, and dyslipidemia compared to the control (receiving no intervention) subjects over a mid-term follow-up period. According to the NNT in our study, bariatric surgery, compared to non-surgical interventions, resulted in the treatment of one out of eight patients with T2DM, five patients with HTN, and five patients with dyslipidemia. The relative risk reductions associated with bariatric surgery were 94, 93, and 55% for the development of T2DM, HTN, and dyslipidemia, respectively.

The metabolic health status in the people living in the MENA region is unsatisfactory due to physical inactivity and unhealthy diets; more than one in every three women are obese in most countries of the region [16]. The MENA region harbored the highest global prevalence of T2DM (12.2%) in 2019 [23]. Over the past three decades, unlike European high-income countries where T2DM rates have been stable, the incidence of this condition has almost doubled in the MENA region, even in its high-income countries [16, 24]. The prevalence of HTN in this region was also higher than the rest of the world between 1975 and 2015 [25]. Accordingly, in recent years, metabolic status and morbid obesity have shown deteriorating and soaring trends among Iranian aging men and women [4]. According to the WHO, only 38% of disease preventive programs in the MENA region have been successfully implemented [13], demanding immediate attention to health sectors in the region to effectively implement preventive strategies and maintain optimal metabolic health.

Bariatric surgery provides a sustainable and beneficial treatment for weight loss and improving obesity-related diseases, especially in regions with the highest burden of metabolic risk factors [8, 12]. In our study, the incidence rates of new-onset T2DM, HTN, and dyslipidemia in the participants undergoing bariatric surgery were 2.9%, 1.8%, and 14.3%, respectively. The incidence of new-onset T2DM after bariatric surgery in previous studies has been reported between 0.4 and 9.7%, which was consistent with our finding [26–28]. In Swedish Obese Subjects, T2DM developed in 392 participants in the control group and in 110 in the bariatric-surgery group, corresponding to incidence rates of 28.4 cases per 1000 person-years and 6.8 cases per 1000 person-years, respectively [29, 30]. Studies on the incidence of new-onset HTN and dyslipidemia after bariatric surgeries are infrequent, reporting ranges from 3.1 to 5.6% and from 2.1 to 6.8%, respectively [22, 26, 31]. In this investigation, the incidence of new-onset comorbidities after bariatric surgery was not significantly different between the types of surgical procedures. On the other hand, the incidence rates of T2DM, HTN, and dyslipidemia in the non-surgical group were 15.0%, 20.4%, and 31.5%, respectively, ringing the alarm bell. These incidence rates of obesity-related diseases observed here in people with severe obesity receiving no therapeutic intervention were clearly higher than those reported in previous studies [22, 26–28, 31, 32], which is a reminder of the unfavorable metabolic status of populations in the MENA region.

Bariatric surgery seems to be effective in preventing obesity-related diseases, including T2DM, HTN, and dyslipidemia. Also, the relative risk reductions associated with bariatric surgery were 94, 93, and 55% for the development of T2DM, HTN, and dyslipidemia, respectively. Similarly, a study in Norway compared the preventive effects of bariatric surgery and pharmaceutical treatments against obesity-related comorbidities, reporting the relative risk reductions of 93, 60, and 70% for T2DM, HTN, and dyslipidemia, respectively [14]. In a recent meta-analysis, the risk reduction rates of T2DM, HTN, and dyslipidemia were reported 61, 64, and 77%, respectively [15]. The preventive effect of bariatric surgery against obesity-associated comorbidities was more pronounced in our study compared to previous studies. This difference can be attributed to the fact that the control group’s participants in our study did not receive any treatment while control subjects in similar studies had been medically managed. As well, various surgical procedures, variable follow-up durations, and the low access of people with severe obesity to health facilities in developing countries compared to developed countries can explain this difference. Based on these findings, bariatric surgery seems to be effective in reducing obesity-related comorbidities and long-term all-cause mortality [15]. Regarding the unsatisfactory metabolic status of severely obese people receiving no therapeutic interventions in our population, it is advisable to implement informative programs for severely obese patients at risk of associated comorbidities for considering bariatric surgeries. It should be noted that recent studies have revealed that some pharmacological treatments, in addition to having a significant influence on weight loss, also have a positive effect on improving glycemic status [33, 34]. In developing countries with limited access to bariatric surgery, clinical studies can be conducted to assess the efficacy of these medications in morbidly obese individuals at high risk of developing T2DM. 

Mechanisms for preventing T2DM after bariatric surgery are probably similar to those behind T2DM remission and can be summarized in four pathways. The first is to alter the secretion of hormones such as glucagon-like peptide 1, gastric inhibitory peptide, ghrelin, and glucagon [35, 36]. The second mechanism is related to the histological and anatomical changes caused by hepatic and pancreatic lipid content changes [37, 38]. Changes at cellular and molecular levels, such as inflammatory markers and gene expression, can be regarded as the third mechanism [39]. Finally, gut microbiome changes can be associated with decreased lipopolysaccharide and lipopolysaccharide-binding protein levels and altered glucose metabolic rate [40]. The remission and prevention of HTN after bariatric surgery can be attributed to weight control and modulation of hormonal mechanisms [41]. The elevation of serum nitric oxide and improvement of endothelial vasomotor function and aortic elasticity are some other explanations noted in previous studies [42–44].

Our study has several strengths and limitations. To the best of our knowledge, our retrospective study on two population-based cohorts with a mid-term follow-up was the first report concerning the preventive effects of bariatric surgery against obesity-related diseases in a developing country in the MENA region. The current study has fourth noteworthy limitations. First, the small number of patients with new-onset comorbidities at the end of the follow-up may reduce the accuracy of the calculated risks. Second, no data was available on the participants’ nutritional and social status and physical activities for the surgery group. Third, lack of treatment control group, which would be considered that the people participating in the TLGS are more conscious of their health status than the overall population. Fourth, T2DM was defined differently in the two groups (in TOTS with HbA1c or FPG or medication; TLGS with 2-hPG or FPG or medication), which was likely to affect the different incidence of T2DM in both groups. 

## Conclusion

Among participants with severe obesity, bariatric surgery was associated with a clinically meaningful reduction in the risk of new-onset T2DM, HTN, and dyslipidemia compared to a control group. The key role of bariatric surgery in reducing the risk of obesity-related comorbidities should be considered in the decision-making process for public health in Iran and other countries in the MENA region. Due to the undesirable metabolic status of the people living in this region and the preventive role of bariatric surgery in developing obesity-related diseases, it is suggested that clinical trials be designed to evaluate the affordability and cost–benefit of bariatric surgery for patients with BMI less than 35 kg/m^2^ in the countries of MENA region.

## Supplementary Information

**Additional file 1: Table S1.** Baseline Characteristics of Study Participants based on comorbidities at baseline.

## Data Availability

The datasets used and analyzed in the current study are available from the corresponding author on reasonable request.

## Abbreviations

BMI: Body mass index
T2DM: Type 2 diabetes mellitus
HTN: Hypertension
MENA: Middle East and North Africa
TLGS: Tehran Lipid, and Glucose Study
TOTS: Tehran Obesity Treatment Study
SG: Sleeve gastrectomy
OAGB: One-anastomosis gastric bypass
RYGB: Roux-en-Y gastric bypass
WHO: World health organization
BP: Blood pressure
FPG: Fasting plasma glucose
HDL: High-density lipoprotein-cholesterol
TC: Total cholesterol
LDL: Low-density lipoprotein-cholesterol
SD: Standard deviation
IQR: Interquartile range
AR: Absolute risk
RR: Relative risk
ARR: Absolute risk reduction
NNT: Number need to treat

## References

[1] Mokdad AH, Marks JS, Stroup DF, Gerberding JL (2004). Actual causes of death in the United States, 2000. JAMA.

[2] Smith KB, Smith MS (2016). Obesity statistics. Prim Care.

[3] Amin R, Kolahi AA, Sohrabi MR (2021). Disparities in obesity prevalence in iranian adults: cross-sectional study using data from the 2016 STEPS Survey. Obes Facts.

[4] Mousapour P, Valizadeh M, Mahdavi M, Saadat N, Barzin M, Azizi F (2020). Trends in the prevalence of severe obesity among tehranian adults: tehran lipid and glucose study, 1999–2017. Arch Iran Med.

[5] Lavie CJ, De Schutter A, Parto P, Jahangir E, Kokkinos P, Ortega FB (2016). Obesity and prevalence of cardiovascular diseases and prognosis-the obesity paradox updated. Prog Cardiovasc Dis.

[6] Whitlock G, Lewington S, Sherliker P, Clarke R, Emberson J, Halsey J (2009). Body-mass index and cause-specific mortality in 900 000 adults: collaborative analyses of 57 prospective studies. Lancet (London, England).

[7] Ryan D, Heaner M (2014). Guidelines (2013) for managing overweight and obesity in adults. Preface to the full report. Obesity (Silver Spring, Md)..

[8] Gloy VL, Briel M, Bhatt DL, Kashyap SR, Schauer PR, Mingrone G (2013). Bariatric surgery versus non-surgical treatment for obesity: a systematic review and meta-analysis of randomised controlled trials. BMJ (Clin Res Ed).

[9] Wilding JPH, Batterham RL, Calanna S, Davies M, Van Gaal LF, Lingvay I (2021). Once-weekly semaglutide in adults with overweight or obesity. N Engl J Med.

[10] Rubino D, Abrahamsson N, Davies M, Hesse D, Greenway FL, Jensen C (2021). Effect of continued weekly subcutaneous semaglutide vs placebo on weight loss maintenance in adults with overweight or obesity: the STEP 4 randomized clinical trial. JAMA.

[11] Davies M, Færch L, Jeppesen OK, Pakseresht A, Pedersen SD, Perreault L (2021). Semaglutide 2·4 mg once a week in adults with overweight or obesity, and type 2 diabetes (STEP 2): a randomised, double-blind, double-dummy, placebo-controlled, phase 3 trial. Lancet (London, England).

[12] O'Brien PE, Hindle A, Brennan L, Skinner S, Burton P, Smith A (2019). Long-term outcomes after bariatric surgery: a systematic review and meta-analysis of weight loss at 10 or more years for all bariatric procedures and a single-centre review of 20-year outcomes after adjustable gastric banding. Obes Surg.

[13] Vitiello A, Angrisani L, Santonicola A, Iovino P, Pilone V, Forestieri P (2019). Bariatric surgery versus lifestyle intervention in class I obesity: 7–10-year results of a retrospective study. World J Surg.

[14] Jakobsen GS, Småstuen MC, Sandbu R, Nordstrand N, Hofsø D, Lindberg M (2018). Association of bariatric surgery vs medical obesity treatment with long-term medical complications and obesity-related comorbidities. JAMA.

[15] Wiggins T, Guidozzi N, Welbourn R, Ahmed AR, Markar SR (2020). Association of bariatric surgery with all-cause mortality and incidence of obesity-related disease at a population level: a systematic review and meta-analysis. PLoS Med.

[16] Azizi F, Hadaegh F, Hosseinpanah F, Mirmiran P, Amouzegar A, Abdi H (2019). Metabolic health in the Middle East and north Africa. Lancet Diabetes Endocrinol.

[17] Welbourn R, Hollyman M, Kinsman R, Dixon J, Liem R, Ottosson J (2019). Bariatric surgery worldwide: baseline demographic description and one-year outcomes from the fourth IFSO Global Registry Report 2018. Obes Surg.

[18] Azizi F, Ghanbarian A, Momenan AA, Hadaegh F, Mirmiran P, Hedayati M (2009). Prevention of non-communicable disease in a population in nutrition transition: Tehran lipid and glucose study phase II. Trials.

[19] Barzin M, Hosseinpanah F, Motamedi MA, Shapoori P, Arian P, Daneshpour MA (2016). Bariatric surgery for morbid obesity: Tehran Obesity Treatment Study (TOTS) rationale and study design. JMIR Res Protocols.

[20] Report of the expert committee on the diagnosis and classification of diabetes mellitus. Diabetes Care. 2003;26(Suppl 1):S5–20. 10.2337/diacare.26.2007.s5 12502614

[21] Mancia G, De Backer G, Dominiczak A, Cifkova R, Fagard R, Germano G (2007). 2007 Guidelines for the Management of Arterial Hypertension: The Task Force for the Management of Arterial Hypertension of the European Society of Hypertension (ESH) and of the European Society of Cardiology (ESC). J Hypertens.

[22] Singh P, Subramanian A, Adderley N, Gokhale K, Singhal R, Bellary S (2020). Impact of bariatric surgery on cardiovascular outcomes and mortality: a population-based cohort study. Br J Surg.

[23] Saeedi P, Petersohn I, Salpea P, Malanda B, Karuranga S, Unwin N (2019). Global and regional diabetes prevalence estimates for 2019 and projections for 2030 and 2045: results from the International Diabetes Federation Diabetes Atlas. Diabetes Res Clin Pract.

[24] Worldwide trends in diabetes since 1980: a pooled analysis of 751 population-based studies with 4.4 million participants. Lancet (London, England). 2016;387(10027):1513–30. 10.1016/S0140-6736(16)00618-8 PMC5081106 27061677

[25] Worldwide trends in blood pressure from 1975 to 2015: a pooled analysis of 1479 population-based measurement studies with 19·1 million participants. Lancet (London, England). 2017;389(10064):37–55. 10.1016/S0140-6736(16)31919-5 PMC5220163 27863813

[26] Pontiroli AE, Zakaria AS, Fanchini M, Osio C, Tagliabue E, Micheletto G (2018). A 23-year study of mortality and development of co-morbidities in patients with obesity undergoing bariatric surgery (laparoscopic gastric banding) in comparison with medical treatment of obesity. Cardiovasc Diabetol.

[27] Thereaux J, Lesuffleur T, Czernichow S, Basdevant A, Msika S, Nocca D (2018). Association between bariatric surgery and rates of continuation, discontinuation, or initiation of antidiabetes treatment 6 years later. JAMA Surg.

[28] Bailly L, Schiavo L, Sebastianelli L, Fabre R, Morisot A, Pradier C (2019). Preventive effect of bariatric surgery on type 2 diabetes onset in morbidly obese inpatients: a national French survey between 2008 and 2016 on 328,509 morbidly obese patients. Surg Obes Rel.

[29] Sjöström L (2013). Review of the key results from the Swedish Obese Subjects (SOS) trial—a prospective controlled intervention study of bariatric surgery. J Intern Med.

[30] Carlsson LM, Peltonen M, Ahlin S, Anveden Å, Bouchard C, Carlsson B (2012). Bariatric surgery and prevention of type 2 diabetes in Swedish obese subjects. N Engl J Med.

[31] Thereaux J, Lesuffleur T, Czernichow S, Basdevant A, Msika S, Nocca D (2019). Multicentre cohort study of antihypertensive and lipid-lowering therapy cessation after bariatric surgery. Br J Surg.

[32] Reges O, Greenland P, Dicker D, Leibowitz M, Hoshen M, Gofer I (2018). Association of bariatric surgery using laparoscopic banding, Roux-en-Y Gastric bypass, or laparoscopic sleeve gastrectomy vs usual care obesity management with all-cause mortality. JAMA.

[33] Rubino DM, Greenway FL, Khalid U, O'Neil PM, Rosenstock J, Sørrig R (2022). Effect of weekly subcutaneous semaglutide vs daily liraglutide on body weight in adults with overweight or obesity without diabetes: the STEP 8 randomized clinical trial. JAMA.

[34] Wadden TA, Bailey TS, Billings LK, Davies M, Frias JP, Koroleva A (2021). Effect of subcutaneous semaglutide vs placebo as an adjunct to intensive behavioral therapy on body weight in adults with overweight or obesity: the STEP 3 randomized clinical trial. JAMA.

[35] Nosso G, Griffo E, Cotugno M, Saldalamacchia G, Lupoli R, Pacini G (2016). Comparative effects of Roux-en-Y gastric bypass and sleeve gastrectomy on glucose homeostasis and incretin hormones in obese type 2 diabetic patients: a one-year prospective study. Hormone Metab Res..

[36] Umeda LM, Silva EA, Carneiro G, Arasaki CH, Geloneze B, Zanella MT (2011). Early improvement in glycemic control after bariatric surgery and its relationships with insulin, GLP-1, and glucagon secretion in type 2 diabetic patients. Obes Surg.

[37] Immonen H, Hannukainen JC, Iozzo P, Soinio M, Salminen P, Saunavaara V (2014). Effect of bariatric surgery on liver glucose metabolism in morbidly obese diabetic and non-diabetic patients. J Hepatol.

[38] Honka H, Koffert J, Hannukainen JC, Tuulari JJ, Karlsson HK, Immonen H (2015). The effects of bariatric surgery on pancreatic lipid metabolism and blood flow. J Clin Endocrinol Metab.

[39] Russel SM, Valle V, Spagni G, Hamilton S, Patel T, Abdukadyrov N (2020). Physiologic mechanisms of type II diabetes mellitus remission following bariatric surgery: a meta-analysis and clinical implications. J Gastrointest Surg.

[40] Clemente-Postigo M, Roca-Rodriguez Mdel M, Camargo A, Ocaña-Wilhelmi L, Cardona F, Tinahones FJ (2015). Lipopolysaccharide and lipopolysaccharide-binding protein levels and their relationship to early metabolic improvement after bariatric surgery. Surg Obes Rel Dis.

[41] Adams ST, Salhab M, Hussain ZI, Miller GV, Leveson SH (2013). Obesity-related hypertension and its remission following gastric bypass surgery—a review of the mechanisms and predictive factors. Blood Press.

[42] Sledzinski T, Sledzinski M, Smolenski RT, Swierczynski J (2010). Increased serum nitric oxide concentration after bariatric surgery—a potential mechanism for cardiovascular benefit. Obes Surg.

[43] Gokce N, Vita JA, McDonnell M, Forse AR, Istfan N, Stoeckl M (2005). Effect of medical and surgical weight loss on endothelial vasomotor function in obese patients. Am J Cardiol.

[44] Ikonomidis I, Mazarakis A, Papadopoulos C, Patsouras N, Kalfarentzos F, Lekakis J (2007). Weight loss after bariatric surgery improves aortic elastic properties and left ventricular function in individuals with morbid obesity: a 3-year follow-up study. J Hypertens.

