# Supplementary material for: The effect of bariatric surgery in comparison with the control group on the prevention of comorbidities in people with severe obesity: a prospective cohort study
Source: BMC Surg. 2022 Jul 28;22:290. doi: 10.1186/s12893-022-01740-7 (PMC9331579; doi:10.1186/s12893-022-01740-7)
Supplement: Supplementary file 1 — Additional file 1: Table S1. Baseline Characteristics of Study Participants based on comorbidities at baseline. [file 12893_2022_1740_MOESM1_ESM.docx]

| **Supplementary Table 1**. Baseline Characteristics of Study Participants based on comorbidities at baseline | | | | | | | | | |
| --- | --- | --- | --- | --- | --- | --- | --- | --- | --- |
|  | **Participants without T2DM at baseline** | | | **Participants without HTN at baseline** | | | **Participants without dyslipidemia at baseline** | | |
| **Characteristic** | **Surgery group**  **N= 272** | **Control group**  **N= 440** | ***P* Value** | **Surgery group**  **N= 224** | **Control group**  **N= 343** | ***P* Value** | **Surgery group**  **N= 230** | **Control group**  **N= 295** | ***P* Value** |
| **Female** n (%) | 225 (82.7) | 339 (77.0) | 0.070 | 186 (83.0) | 273 (79.6) | 0.307 | 199 (86.5) | 247 (83.7) | 0.375 |
| **Age** (year) | 37.8 ± 10.4 | 48.2 ± 12.6 | <0.001 | 37.3 ± 10.6 | 45.6 ± 12.6 | <0.001 | 39.5 ± 11.3 | 47.6 ± 12.3 | <0.001 |
| **Weight** (kg) | 119.2 ± 20.8 | 96.6 ± 15.6 | <0.001 | 118.8 ± 20.7 | 96.1 ± 14.9 | <0.001 | 118.8 ± 19.2 | 96.5 ± 15.7 | <0.001 |
| **BMI** (kg/m^2^) | 44.6 ± 5.9 | 38.4 ± 3.5 | <0.001 | 44.7 ± 6.0 | 38.3 ± 3.4 | <0.001 | 45.1 ± 6.2 | 38.8 ± 3.9 | <0.001 |
| **BMI < 40**, n (%) | 48 (17.6) | 327 (74.3) | <0.001 | 46 (20.5) | 261 (76.1) | <0.001 | 45 (19.6) | 206 (69.8) | <0.001 |
| **BMI 40–50**, n (%) | 183 (67.3) | 110 (25.0) |  | 141 (62.9) | 80 (23.3) |  | 144 (62.6) | 85 (28.8) |  |
| **BMI > 50**, n (%) | 41 (15.1) | 3 (0.7) |  | 37 (16.5) | 2 (0.6) |  | 41 (17.8) | 4 (1.4) |  |
| **Waist circumference** (cm) | 123.0 ± 13.1 | 113.0 ± 9.9 | <0.001 | 123.1 ± 13.6 | 112.3 ± 9.9 | <0.001 | 122.3 ± 15.3 | 113.4 ± 10.3 | <0.001 |
| **Hip circumference** (cm) | 135.7 ± 12.4 | 116.1 ± 8.0 | <0.001 | 135.2 ± 12.3 | 116.0 ± 7.8 | <0.001 | 136.8 ± 11.9 | 116.9 ± 7.7 | <0.001 |
| **FPG** (mg/dl) | 96.8 ± 10.3 | 96.8 ± 9.3 | 0.968 | 106.2 ± 33.1 | 101.8 ± 20.9 | 0.076 | 106.8 ± 35.0 | 100.8 ± 16.2 | 0.016 |
| **HbA1c** % | 5.3 (5.1-5.7) | NA | NA | 5.4 (5.1-5.8) | NA | NA | 5.4 (5.1-5.9) | NA | NA |
| **2-hPG** (mg/dl) | NA | 116.6 ± 29.9 | NA | NA | 119.0 ± 35.8 | NA | NA | 123.5 ± 42.8 | NA |
| **Impaired fasting glucose** n (%) | 129 (47.4) | 185 (42.0) | 0.160 | 72 (44.4) | 105 (37.8) | 0.168 | 75 (42.1) | 93 (38.8) | 0.485 |
| **T2DM** n (%) | 0 | 0 | NA | 44 (22.0) | 58 (17.8) | 0.243 | 45 (20.3) | 47 (16.8) | 0.325 |
| **Systolic BP** (mm Hg) | 123.1 ± 12.8 | 125.5 ± 18.7 | 0.046 | 118.1 ± 8.8 | 115.6 ± 11.0 | 0.005 | 122.9 ± 13.8 | 124.5 ± 18.9 | 0.279 |
| **Diastolic BP** (mm Hg) | 79.2 ± 8.1 | 82.2 ± 10.7 | <0.001 | 76.0 ± 7.0 | 77.0 ± 7.3 | 0.134 | 79.1 ± 9.1 | 81.8 ± 11.1 | 0.003 |
| **HTN**, n (%) | 66 (25.9) | 190 (43.4) | <0.001 | 0 | 0 | NA | 60 (27.8) | 111 (37.8) | 0.023 |
| **Total cholesterol** (mg/dl) | 192.8 ± 35.5 | 200.0 ± 38.2 | 0.014 | 192.9 ± 37.1 | 197.0 ± 36.8 | 0.205 | 180.1 ± 27.0 | 191.8 ± 27.3 | <0.001 |
| **Triglycerides** (mg/dl) | 135.0 (100-184) | 148.0 (113-194) | 0.116 | 133.0 (100-194) | 145.0 (107-189) | 0.336 | 118.0 (91-143) | 124.0 (96-153) | 0.231 |
| **HDL cholesterol** (mg/dl) | 49.1 ± 11.8 | 48.0 ± 11.6 | 0.240 | 50.0 ± 12.2 | 48.2 ± 11.4 | 0.084 | 49.1 ± 11.8 | 48.0 ± 11.6 | 0.364 |
| **LDL cholesterol** (mg/dl) | 112.1 ± 30.9 | 118.9 ± 32.8 | 0.007 | 110.9 ± 32.6 | 117.2 ± 32.4 | 0.026 | 102.7 ± 26.0 | 114.3 ± 25.1 | <0.001 |
| **Dyslipidemia** n (%) | 131 (50.4) | 225 (51.3) | 0.824 | 98 (47.1) | 159 (46.4) | 0.862 | 0 | 0 | NA |
| **Never smoking** n (%) | 215 (83.7) | 387 (88.2) | 0.011 | 167 (78.8) | 301 (89.1) | 0.002 | 182 (83.9) | 258 (88.4) | 0.241 |
| **Family history of T2DM** n (%) | 96 (48.7) | 44 (10.2) | <0.001 | 81 (48.5) | 48 (14.2) | <0.001 | 78 (46.4) | 38 (13.0) | <0.001 |
| BMI, body mass index; FPG, fasting plasma glucose; 2-hPG, 2-hours plasma glucose; T2DM, type 2 diabetes mellitus; BP, blood pressure; HTN, Hypertension.  Data are presented as mean ±SD or n (%) expect triglycerides which are presented as median (IQ 25-75). | | | | | | | | | |
